# Inter­molecular hydrogen bonding in isostructural pincer complexes [OH-(^*t*-Bu^POCOP^*t*-Bu^)*M*Cl] (*M* = Pd and Pt)

**DOI:** 10.1107/S2056989019008491

**Published:** 2019-06-21

**Authors:** Markus Joksch, Anke Spannenberg, Torsten Beweries

**Affiliations:** a Leibniz-Institut für Katalyse e. V. an der Universität Rostock, Albert-Einstein-Str. 29a, 18059 Rostock, Germany

**Keywords:** pincer complexes, palladium, platinum, hydrogen bonding, crystal structure

## Abstract

In the crystal structure of the isostructural title compounds, namely {2,6-bis­[(di-*tert*-butyl­phosphan­yl)­oxy]-4-hy­droxy­phen­yl}chlorido­palladium(II), [Pd(C_22_H_39_O_3_P_2_)Cl], **1**, and {2,6-bis­[(di-*tert*-butyl­phosphan­yl)­oxy]-4-hy­droxy­phen­yl}chlorido­platinum(II), [Pt(C_22_H_39_O_3_P_2_)Cl], **2**, the metal centres are coordinated in a distorted square-planar fashion by the POCOP pincer fragment and the chloride ligand. Both complexes form strong hydrogen-bonded chain structures through an inter­action of the OH group in the 4-position of the aromatic POCOP backbone with the halide ligand.

## Abstract 2

In the crystal structure of the isostructural title compounds [OH-(^*t*-Bu^POCOP^*t*-Bu^)PdCl] and [OH-(^*t*-Bu^POCOP^*t*-Bu^)PtCl] the *M* ^II^ centres are coordinated in a distorted square-planar fashion by the pincer and the chloride ligand.

## Chemical context   

Since their discovery by Shaw and van Koten in the 1970s (Moulton & Shaw, 1976 ▸; van Koten *et al.*, 1978 ▸), pincer complexes have received considerable attention in organometallic chemistry and homogeneous catalysis because of their wide applicability for a broad range of stoichiometric and catalytic bond-activation reactions (*e.g*. Szabo & Wendt, 2014 ▸; Valdés *et al.*, 2018 ▸). Modification of the pincer scaffold allows for fine-tuning of the steric and electronic properties that directly influence the reactivity (Peris & Crabtree, 2018 ▸). As a consequence, a plethora of transition metal complexes that possess neutral and anionic tridentate pincer ligands with many different combinations of donor atoms have been described. Substitution of the pincer backbone with suitable polar groups provides an excellent opportunity for the introduction of anchoring sites that can be attached covalently to a heterogeneous support (Rimoldi *et al.*, 2016 ▸). In this context, hy­droxy­lation of the aromatic ring of a POCOP ligand is a straightforward approach and the ligand precursor phloro­glucinol is a readily available compound that can be converted into the corresponding ligand using standard methodologies (Göttker-Schnetmann *et al.*, 2004 ▸; Garcia-Eleno *et al.*, 2015 ▸). This polar functionality can engage in non-covalent inter­actions with ubiquitous metal-halide fragments. An example for this phenomenon that includes halide–halide inter­actions was reported recently by Whitwood, Brammer and Perutz, who studied inter­molecular halogen bonding of a series of nickel(II) fluoride complexes (Thangavadivale *et al.*, 2018 ▸). For a recent review article on the application of pincer complexes, see Valdés *et al.* (2018 ▸).
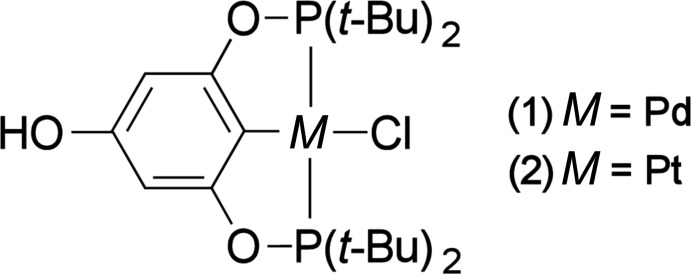


## Structural commentary   

**Figure 1:**
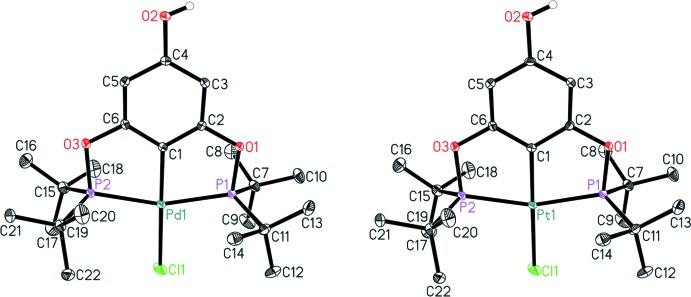
Mol­ecular structure of complexes **1** (left) and **2** (right), with displacement ellipsoids drawn at the 30% probability level. Hydrogen atoms (except of the OH group) are omitted for clarity.

Complexes **1** and **2** are isomorphous and both crystallize in the monoclinic space group *P*2_1_/*n* with one mol­ecule in the asymmetric unit. The mol­ecular structures (Fig. 1 ▸) show the metal(II) centres in a distorted square-planar coordination environment. The distortion is evidenced by the P—*M*—P angles that strongly deviate from 180° [**1**: 159.768 (12), **2**: 160.676 (17)°]. The *M*—Cl [**1**: 2.3871 (4), **2**: 2.3907 (5) Å] and *M*—P bonds [**1**: 2.2880 (3), 2.2918 (3); **2**: 2.2781 (5), 2.2796 (5) Å] are in the expected ranges and are in line with values found in previous examples for Pd and Pt PCP pincer complexes (*e.g.* Bolliger *et al.*, 2007 ▸; Joksch *et al.*, 2017 ▸, 2018 ▸). As can be seen from the structural data, variation of the metal centre does not affect the structural features of the pincer complex. Complexes **1** and **2** are isostructural to the di­chloro­ethane solvate of a similar nickel complex (Garcia-Eleno *et al.*, 2015 ▸).

## Supra­molecular features   

**Figure 2:**
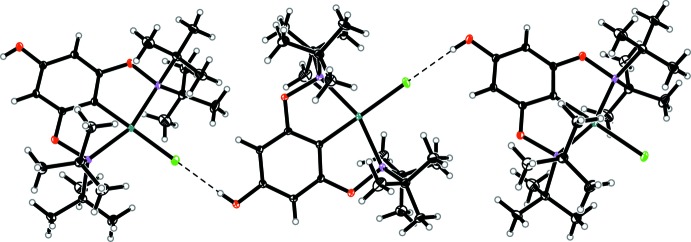
Inter­molecular hydrogen bonds (depicted as dashed lines) in complex **1**. Displacement ellipsoids are drawn at the 30% probability level.

In both complexes, the OH group in the 4-position of the POCOP ligand shows pronounced inter­molecular hydrogen bonding to the chloride ligand (Tables 1 ▸ and 2 ▸), thus resulting in the formation of infinite chain structures along [101] (Figs. 2 ▸ and 3 ▸). A dihedral angle of 31.38 (6)° in **1** and 31.74 (9)° in **2** between the planes of the aryl rings of neighbouring pincer complexes involved in hydrogen bonding was observed.

## Database survey   

A search of the Cambridge Structural Database (CSD, Version 5.40, May 2019 update; Groom *et al.*, 2016 ▸) for Pd and Pt POCOP halide complexes with aromatic ligand backbones resulted in 58 hits. Similar Pd and Pt pincer complexes without the OH group in the 4-position have been reported by our group (Joksch *et al.*, 2017 ▸, 2018 ▸). Related complexes have been described, *e.g*. by Frech and co-workers (Bolliger *et al.*, 2007 ▸), Jensen and co-workers (Morales-Morales *et al.*, 2000 ▸; Wang *et al.*, 2006 ▸), Wendt and co-workers (Johnson *et al.*, 2013 ▸) or Milstein and co-workers (Vuzman *et al.*, 2007 ▸).

## Synthesis and crystallization   

**Complex 1:** Pd(MeCN)Cl_2_ (261 mg, 1.01 mmol) and the ligand precursor 3,5-bis­[(di-*tert*-butyl­phosphan­yl)­oxy]phenol (501 mg, 1.21 mmol) were dissolved in 20 mL of toluene and the mixture was heated at 388 K for two days, resulting in a yellow solution. Upon slow cooling, complex **1** precipitated as a pale-yellow solid, which was isolated by filtration and washed with cold toluene. Colourless crystals suitable for X-ray analysis were obtained from a saturated toluene solution at 195 K, yield: 216 mg (39%). ^1^H NMR (300.13 MHz, 295 K, toluene-*d*_8_): 5.98 (*s*, 2H, *m*-C*H*), 3.92 (*br s*, 1H, O*H*), 1.34 ppm (*vt*, 36H, *t*-Bu). ^13^C NMR (75.48 MHz, 295 K, toluene-*d*_8_, assigned by ^1^H-^13^C-HMBC): 167.4 [*C*-OP(*t*-Bu)_2_], 157.2 (*C*—OH), 121.4 (Pd—*C*), 94.4 (*m*—*C*H), 39.5 [*C*(CH_3_)_3_], 27.6 [C(*C*H_3_)_3_]. ^31^P NMR (121.50 MHz, 295 K, toluene-*d*_8_): 193.5 ppm. Analysis calculated for C_22_H_39_ClO_3_P_2_Pd: C, 47.58; H, 7.08. Found: C, 47.43; H, 7.13. MS (CI positive, *iso*-butane): *m*/*z* 554 [*M*]^+^.

**Complex 2:** Pt(cod)Cl_2_ (147 mg, 0.39 mmol) and the ligand precursor 3,5-bis­[(di-*tert*-butyl­phosphan­yl)­oxy]phenol (195 mg, 1.47 mmol) were dissolved in 15 mL of toluene and the mixture was heated at 388 K for 16 h, resulting in a colourless solution. After cooling to room temperature, the solvent was removed in vacuum and the residue was washed with *n*-hexane to yield complex **2** as a colourless solid. Crystals suitable for X-ray analysis were obtained by slow cooling of a hot saturated toluene solution to room temperature. Yield: 214 mg (85%). ^1^H NMR (400.13 MHz, 297 K, toluene-*d*_8_): 6.03 (*t*, *J* = 7.53 Hz, 2H, *m*-C*H*), 4.10 (*br s*, 1H, O*H*), 1.33 (*vt*, 36H, *t*-Bu). ^13^C NMR (100.63 MHz, 297 K, toluene-*d*_8_): 165.8 [*C*—OP(*t*-Bu)_2_], 156.5 (*C*—OH), 112.5 (Pt—*C*), 94.2 (*m*—*C*H), 40.6 [*C*(CH_3_)_3_], 27.6 [C(*C*H_3_)_3_]. ^31^P NMR (161.98 MHz, 297 K, toluene-*d*_8_): 178.1. Analysis calculated for C_22_H_39_ClO_3_P_2_Pt: C, 41.03; H, 6.10. Found: C, 41.17; H, 5.99. MS (CI positive, *iso*-butane): *m*/*z* 644 [*M*]^+^, 608 [*M* - Cl]^+^.

## Refinement   

Crystal data, data collection and structure refinement details are summarized in Table 3 ▸. Hydrogen atoms attached to oxygen could be found in a difference-Fourier map and were refined freely. All other H atoms were placed in idealized positions with *d*(C—H) = 0.95 Å (CH), 0.98 Å (CH_3_) and refined using a riding model with *U*_iso_(H) fixed at 1.2*U*_eq_(C) for CH and 1.5*U*_eq_(C) for CH_3_. A rotating model was used for the methyl groups.

## Supplementary Material

Crystal structure: contains datablock(s) 1, 2, New_Global_Publ_Block. DOI: 10.1107/S2056989019008491/rz5258sup1.cif

Structure factors: contains datablock(s) 1. DOI: 10.1107/S2056989019008491/rz52581sup2.hkl

Structure factors: contains datablock(s) 2. DOI: 10.1107/S2056989019008491/rz52582sup3.hkl

CCDC references: 1923006, 1923005

Additional supporting information:  crystallographic information; 3D view; checkCIF report

## Figures and Tables

**Figure 3:**
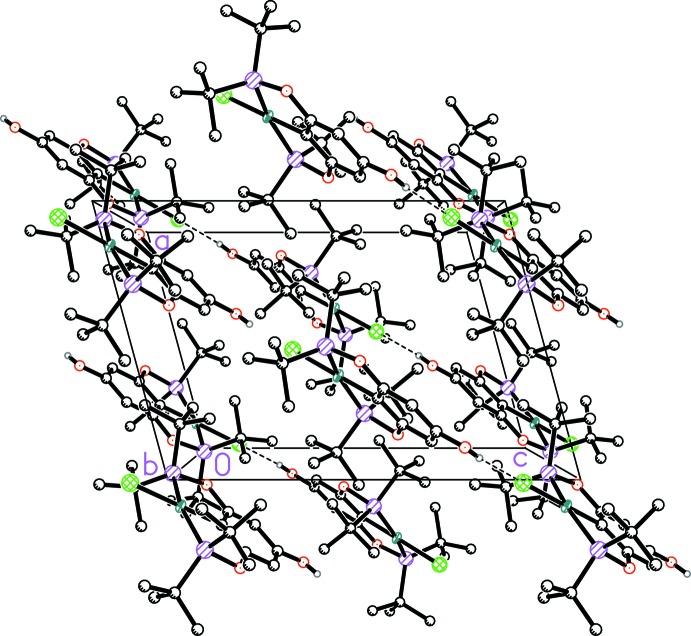
Perspective packing diagram of complex **1** viewed down the *b* axis showing the hydrogen bonds as dashed lines. Hydrogen atoms (except of OH groups) are omitted for clarity.

**Table 1:** Hydrogen-bond geometry (Å, °) for **1** [Chem scheme1]

*D*—H⋯*A*	*D*—H	H⋯*A*	*D*⋯*A*	*D*—H⋯*A*
O2—H2⋯Cl1^i^	0.79 (2)	2.37 (2)	3.1545 (11)	174.2 (19)

Symmetry code: (i) 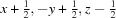.

**Table 2:** Hydrogen-bond geometry (Å, °) for **2** [Chem scheme1]

*D*—H⋯*A*	*D*—H	H⋯*A*	*D*⋯*A*	*D*—H⋯*A*
O2—H2⋯Cl1^i^	0.82 (3)	2.38 (3)	3.1874 (16)	170 (3)

Symmetry code: (i) 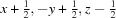.

**Table 3:** Experimental details

	**1**	**2**
Crystal data		
Chemical formula	[Pd(C_22_H_39_O_3_P_2_)Cl]	[Pt(C_22_H_39_O_3_P_2_)Cl]
*M*_r_	555.32	644.01
Crystal system, space group	Monoclinic, *P*2_1_/*n*	Monoclinic, *P*2_1_/*n*
Temperature (K)	150	150
*a*, *b*, *c* (Å)	9.7678 (5), 20.1652 (10), 13.9656 (7)	9.7722 (8), 20.1562 (16), 13.9699 (11)
β (°)	105.1743 (8)	105.1634 (13)
*V* (Å^3^)	2654.9 (2)	2655.9 (4)
*Z*	4	4
Radiation type	Mo *K*α	Mo *K*α
μ (mm^−1^)	0.94	5.52
Crystal size (mm)	0.37 × 0.36 × 0.36	0.34 × 0.21 × 0.15

Data collection		
Diffractometer	Bruker APEXII CCD	Bruker APEXII CCD
Absorption correction	Multi-scan (*SADABS*; Bruker, 2014 ▸)	Multi-scan (*SADABS*; Bruker, 2014 ▸)
*T*_min_, *T*_max_	0.68, 0.73	0.34, 0.50
No. of measured, independent and observed [*I* > 2σ(*I*)] reflections	57317, 6411, 6166	25029, 6413, 5959
*R*_int_	0.018	0.023
(sin θ/λ)_max_ (Å^−1^)	0.661	0.661

Refinement		
*R*[*F* ^2^ > 2σ(*F* ^2^)], *wR*(*F* ^2^), *S*	0.018, 0.046, 1.08	0.016, 0.037, 1.02
No. of reflections	6411	6413
No. of parameters	278	278
H-atom treatment	H atoms treated by a mixture of independent and constrained refinement	H atoms treated by a mixture of independent and constrained refinement
Δρ_max_, Δρ_min_ (e Å^−3^)	0.49, −0.46	0.87, −0.84

Computer programs: *APEX2* and *SAINT* (Bruker, 2014 ▸), *SHELXS97* and *XP* in *SHELXTL* (Sheldrick, 2008 ▸), *SHELXL2014/7* (Sheldrick, 2015 ▸) and *publCIF* (Westrip, 2010 ▸).

## supplementary crystallographic information

### {2,6-Bis[(di-tert-butylphosphanyl)oxy]-4-hydroxyphenyl}chloridopalladium(II) (1) Crystal data

**Table:**

[Pd(C_22_H_39_O_3_P_2_)Cl]	*F*(000) = 1152
*M**_r_* = 555.32	*D*_x_ = 1.389 Mg m^−^^3^
Monoclinic, *P*2_1_/*n*	Mo *K*α radiation, λ = 0.71073 Å
*a* = 9.7678 (5) Å	Cell parameters from 9082 reflections
*b* = 20.1652 (10) Å	θ = 2.4–30.5°
*c* = 13.9656 (7) Å	µ = 0.94 mm^−^^1^
β = 105.1743 (8)°	*T* = 150 K
*V* = 2654.9 (2) Å^3^	Prism, colourless
*Z* = 4	0.37 × 0.36 × 0.36 mm

### {2,6-Bis[(di-tert-butylphosphanyl)oxy]-4-hydroxyphenyl}chloridopalladium(II) (1) Data collection

**Table:**

Bruker APEXII CCD diffractometer	6411 independent reflections
Radiation source: fine-focus sealed tube	6166 reflections with *I* > 2σ(*I*)
Detector resolution: 8.3333 pixels mm^-1^	*R*_int_ = 0.018
φ and ω scans	θ_max_ = 28.0°, θ_min_ = 1.8°
Absorption correction: multi-scan (SADABS; Bruker, 2014)	*h* = −12→12
*T*_min_ = 0.68, *T*_max_ = 0.73	*k* = −26→26
57317 measured reflections	*l* = −18→18

### {2,6-Bis[(di-tert-butylphosphanyl)oxy]-4-hydroxyphenyl}chloridopalladium(II) (1) Refinement

**Table:**

Refinement on *F*^2^	0 restraints
Least-squares matrix: full	Hydrogen site location: mixed
*R*[*F*^2^ > 2σ(*F*^2^)] = 0.018	H atoms treated by a mixture of independent and constrained refinement
*wR*(*F*^2^) = 0.046	*w* = 1/[σ^2^(*F*_o_^2^) + (0.0197*P*)^2^ + 1.5724*P*] where *P* = (*F*_o_^2^ + 2*F*_c_^2^)/3
*S* = 1.08	(Δ/σ)_max_ < 0.001
6411 reflections	Δρ_max_ = 0.49 e Å^−^^3^
278 parameters	Δρ_min_ = −0.45 e Å^−^^3^

### {2,6-Bis[(di-tert-butylphosphanyl)oxy]-4-hydroxyphenyl}chloridopalladium(II) (1) Special details

**Table:**

Geometry. All esds (except the esd in the dihedral angle between two l.s. planes) are estimated using the full covariance matrix. The cell esds are taken into account individually in the estimation of esds in distances, angles and torsion angles; correlations between esds in cell parameters are only used when they are defined by crystal symmetry. An approximate (isotropic) treatment of cell esds is used for estimating esds involving l.s. planes.

### {2,6-Bis[(di-tert-butylphosphanyl)oxy]-4-hydroxyphenyl}chloridopalladium(II) (1) Fractional atomic coordinates and isotropic or equivalent isotropic displacement parameters (Å^2^)

**Table:**

	*x*	*y*	*z*	*U*_iso_*/*U*_eq_	
C1	0.21967 (12)	0.29775 (6)	−0.06646 (9)	0.0158 (2)	
C2	0.32101 (13)	0.27370 (6)	−0.11161 (9)	0.0157 (2)	
C3	0.38045 (13)	0.31168 (6)	−0.17336 (9)	0.0168 (2)	
H3	0.4473	0.2932	−0.2044	0.020*	
C4	0.33893 (13)	0.37773 (6)	−0.18831 (9)	0.0174 (2)	
C5	0.23941 (13)	0.40494 (6)	−0.14407 (9)	0.0187 (2)	
H5	0.2125	0.4502	−0.1537	0.022*	
C6	0.18083 (12)	0.36393 (6)	−0.08548 (9)	0.0165 (2)	
C7	0.20805 (14)	0.09712 (7)	−0.10482 (10)	0.0232 (3)	
C8	0.11293 (18)	0.13104 (9)	−0.19722 (12)	0.0396 (4)	
H8A	0.1715	0.1584	−0.2289	0.059*	
H8B	0.0431	0.1591	−0.1775	0.059*	
H8C	0.0636	0.0972	−0.2440	0.059*	
C9	0.11526 (19)	0.05436 (9)	−0.05637 (14)	0.0406 (4)	
H9A	0.0636	0.0218	−0.1044	0.061*	
H9B	0.0474	0.0827	−0.0348	0.061*	
H9C	0.1754	0.0313	0.0012	0.061*	
C10	0.31748 (17)	0.05426 (7)	−0.13615 (13)	0.0325 (3)	
H10A	0.3734	0.0297	−0.0786	0.049*	
H10B	0.3805	0.0826	−0.1625	0.049*	
H10C	0.2689	0.0229	−0.1875	0.049*	
C11	0.44379 (14)	0.14362 (7)	0.08081 (10)	0.0227 (3)	
C12	0.40374 (18)	0.08715 (8)	0.14186 (12)	0.0351 (3)	
H12A	0.4794	0.0811	0.2030	0.053*	
H12B	0.3911	0.0461	0.1030	0.053*	
H12C	0.3151	0.0982	0.1586	0.053*	
C13	0.57549 (15)	0.12576 (8)	0.04517 (12)	0.0315 (3)	
H13A	0.6557	0.1171	0.1027	0.047*	
H13B	0.5989	0.1628	0.0068	0.047*	
H13C	0.5557	0.0861	0.0033	0.047*	
C14	0.47797 (16)	0.20604 (8)	0.14625 (11)	0.0300 (3)	
H14A	0.3977	0.2163	0.1736	0.045*	
H14B	0.4951	0.2435	0.1061	0.045*	
H14C	0.5629	0.1980	0.2006	0.045*	
C15	−0.16995 (14)	0.34028 (7)	−0.02248 (11)	0.0246 (3)	
C16	−0.22961 (16)	0.40832 (8)	−0.06139 (13)	0.0354 (3)	
H16A	−0.3313	0.4043	−0.0934	0.053*	
H16B	−0.1806	0.4245	−0.1097	0.053*	
H16C	−0.2150	0.4396	−0.0060	0.053*	
C17	−0.24584 (17)	0.31354 (9)	0.05250 (14)	0.0405 (4)	
H17A	−0.3467	0.3073	0.0197	0.061*	
H17B	−0.2356	0.3453	0.1071	0.061*	
H17C	−0.2038	0.2710	0.0787	0.061*	
C18	−0.19456 (18)	0.29240 (10)	−0.11087 (14)	0.0428 (4)	
H18A	−0.1665	0.2475	−0.0867	0.064*	
H18B	−0.1376	0.3065	−0.1555	0.064*	
H18C	−0.2952	0.2926	−0.1468	0.064*	
C19	0.08541 (15)	0.38393 (7)	0.15092 (10)	0.0228 (3)	
C20	0.24411 (17)	0.39741 (9)	0.16379 (13)	0.0383 (4)	
H20A	0.2846	0.4180	0.2286	0.057*	
H20B	0.2558	0.4273	0.1112	0.057*	
H20C	0.2931	0.3555	0.1598	0.057*	
C21	0.00919 (17)	0.44979 (7)	0.15343 (11)	0.0291 (3)	
H21A	−0.0898	0.4413	0.1529	0.044*	
H21B	0.0122	0.4762	0.0952	0.044*	
H21C	0.0562	0.4741	0.2138	0.044*	
C22	0.0681 (2)	0.33840 (8)	0.23496 (11)	0.0363 (4)	
H22A	0.1114	0.3593	0.2991	0.054*	
H22B	0.1148	0.2959	0.2309	0.054*	
H22C	−0.0330	0.3309	0.2286	0.054*	
Cl1	0.03724 (5)	0.17138 (2)	0.12263 (3)	0.03930 (10)	
O1	0.36634 (9)	0.20853 (4)	−0.09447 (7)	0.01852 (17)	
O2	0.39346 (11)	0.41889 (5)	−0.24683 (8)	0.0243 (2)	
O3	0.07741 (9)	0.39017 (4)	−0.04466 (7)	0.01986 (18)	
P1	0.28824 (3)	0.16660 (2)	−0.02188 (2)	0.01575 (6)	
P2	0.02457 (3)	0.33937 (2)	0.03141 (2)	0.01631 (6)	
Pd1	0.13707 (2)	0.24105 (2)	0.02025 (2)	0.01624 (3)	
H2	0.435 (2)	0.3971 (10)	−0.2769 (15)	0.039 (5)*	

### {2,6-Bis[(di-tert-butylphosphanyl)oxy]-4-hydroxyphenyl}chloridopalladium(II) (1) Atomic displacement parameters (Å^2^)

**Table:**

	*U*^11^	*U*^22^	*U*^33^	*U*^12^	*U*^13^	*U*^23^
C1	0.0166 (5)	0.0163 (5)	0.0162 (5)	0.0017 (4)	0.0072 (4)	0.0020 (4)
C2	0.0173 (5)	0.0139 (5)	0.0161 (5)	0.0029 (4)	0.0049 (4)	0.0016 (4)
C3	0.0168 (5)	0.0179 (5)	0.0179 (5)	0.0027 (4)	0.0082 (4)	0.0014 (4)
C4	0.0193 (5)	0.0170 (5)	0.0179 (5)	0.0004 (4)	0.0082 (4)	0.0031 (4)
C5	0.0218 (6)	0.0143 (5)	0.0220 (6)	0.0030 (4)	0.0094 (5)	0.0028 (4)
C6	0.0160 (5)	0.0177 (5)	0.0175 (5)	0.0028 (4)	0.0072 (4)	−0.0006 (4)
C7	0.0253 (6)	0.0210 (6)	0.0261 (6)	−0.0039 (5)	0.0113 (5)	−0.0061 (5)
C8	0.0368 (8)	0.0459 (9)	0.0296 (8)	0.0011 (7)	−0.0031 (6)	−0.0098 (7)
C9	0.0457 (9)	0.0371 (9)	0.0478 (10)	−0.0214 (7)	0.0282 (8)	−0.0155 (7)
C10	0.0361 (8)	0.0236 (7)	0.0432 (9)	−0.0029 (6)	0.0199 (7)	−0.0129 (6)
C11	0.0245 (6)	0.0212 (6)	0.0213 (6)	0.0024 (5)	0.0041 (5)	0.0058 (5)
C12	0.0422 (9)	0.0301 (8)	0.0312 (8)	0.0009 (6)	0.0065 (7)	0.0154 (6)
C13	0.0246 (7)	0.0330 (8)	0.0351 (8)	0.0087 (6)	0.0047 (6)	0.0049 (6)
C14	0.0317 (7)	0.0327 (7)	0.0228 (7)	−0.0025 (6)	0.0022 (6)	−0.0009 (6)
C15	0.0180 (6)	0.0280 (7)	0.0287 (7)	−0.0009 (5)	0.0078 (5)	−0.0009 (5)
C16	0.0223 (7)	0.0364 (8)	0.0455 (9)	0.0061 (6)	0.0051 (6)	0.0065 (7)
C17	0.0279 (8)	0.0462 (9)	0.0537 (10)	−0.0051 (7)	0.0218 (7)	0.0081 (8)
C18	0.0291 (8)	0.0500 (10)	0.0424 (9)	0.0002 (7)	−0.0030 (7)	−0.0173 (8)
C19	0.0275 (6)	0.0208 (6)	0.0204 (6)	0.0026 (5)	0.0069 (5)	−0.0027 (5)
C20	0.0272 (7)	0.0430 (9)	0.0407 (9)	−0.0008 (7)	0.0015 (6)	−0.0151 (7)
C21	0.0411 (8)	0.0198 (6)	0.0289 (7)	0.0050 (6)	0.0133 (6)	−0.0036 (5)
C22	0.0612 (11)	0.0277 (7)	0.0209 (7)	0.0105 (7)	0.0125 (7)	0.0021 (6)
Cl1	0.0649 (3)	0.02367 (16)	0.0452 (2)	−0.00300 (16)	0.0427 (2)	0.00352 (15)
O1	0.0233 (4)	0.0142 (4)	0.0219 (4)	0.0054 (3)	0.0127 (4)	0.0047 (3)
O2	0.0318 (5)	0.0179 (4)	0.0308 (5)	0.0036 (4)	0.0215 (4)	0.0065 (4)
O3	0.0224 (4)	0.0166 (4)	0.0256 (4)	0.0048 (3)	0.0152 (4)	0.0032 (3)
P1	0.01950 (14)	0.01287 (13)	0.01668 (14)	0.00141 (11)	0.00791 (11)	0.00152 (10)
P2	0.01767 (14)	0.01587 (14)	0.01803 (14)	0.00131 (11)	0.00938 (11)	−0.00007 (11)
Pd1	0.02072 (5)	0.01407 (5)	0.01727 (5)	0.00096 (3)	0.01092 (4)	0.00110 (3)

### {2,6-Bis[(di-tert-butylphosphanyl)oxy]-4-hydroxyphenyl}chloridopalladium(II) (1) Geometric parameters (Å, º)

**Table:**

C1—C2	1.3926 (16)	C14—H14A	0.9800
C1—C6	1.3937 (16)	C14—H14B	0.9800
C1—Pd1	1.9841 (12)	C14—H14C	0.9800
C2—O1	1.3873 (14)	C15—C17	1.531 (2)
C2—C3	1.3891 (16)	C15—C16	1.533 (2)
C3—C4	1.3920 (17)	C15—C18	1.536 (2)
C3—H3	0.9500	C15—P2	1.8516 (14)
C4—O2	1.3670 (14)	C16—H16A	0.9800
C4—C5	1.3936 (17)	C16—H16B	0.9800
C5—C6	1.3878 (17)	C16—H16C	0.9800
C5—H5	0.9500	C17—H17A	0.9800
C6—O3	1.3881 (14)	C17—H17B	0.9800
C7—C10	1.5250 (19)	C17—H17C	0.9800
C7—C9	1.531 (2)	C18—H18A	0.9800
C7—C8	1.539 (2)	C18—H18B	0.9800
C7—P1	1.8557 (13)	C18—H18C	0.9800
C8—H8A	0.9800	C19—C21	1.5274 (18)
C8—H8B	0.9800	C19—C22	1.534 (2)
C8—H8C	0.9800	C19—C20	1.537 (2)
C9—H9A	0.9800	C19—P2	1.8512 (13)
C9—H9B	0.9800	C20—H20A	0.9800
C9—H9C	0.9800	C20—H20B	0.9800
C10—H10A	0.9800	C20—H20C	0.9800
C10—H10B	0.9800	C21—H21A	0.9800
C10—H10C	0.9800	C21—H21B	0.9800
C11—C12	1.5334 (19)	C21—H21C	0.9800
C11—C13	1.539 (2)	C22—H22A	0.9800
C11—C14	1.540 (2)	C22—H22B	0.9800
C11—P1	1.8545 (13)	C22—H22C	0.9800
C12—H12A	0.9800	Cl1—Pd1	2.3871 (4)
C12—H12B	0.9800	O1—P1	1.6521 (9)
C12—H12C	0.9800	O2—H2	0.79 (2)
C13—H13A	0.9800	O3—P2	1.6527 (9)
C13—H13B	0.9800	P1—Pd1	2.2880 (3)
C13—H13C	0.9800	P2—Pd1	2.2918 (3)
			
C2—C1—C6	115.95 (11)	C16—C15—C18	108.22 (13)
C2—C1—Pd1	121.76 (9)	C17—C15—P2	110.34 (11)
C6—C1—Pd1	122.29 (9)	C16—C15—P2	114.03 (10)
O1—C2—C3	117.58 (10)	C18—C15—P2	104.39 (10)
O1—C2—C1	118.97 (10)	C15—C16—H16A	109.5
C3—C2—C1	123.44 (11)	C15—C16—H16B	109.5
C2—C3—C4	117.90 (11)	H16A—C16—H16B	109.5
C2—C3—H3	121.0	C15—C16—H16C	109.5
C4—C3—H3	121.0	H16A—C16—H16C	109.5
O2—C4—C3	121.80 (11)	H16B—C16—H16C	109.5
O2—C4—C5	116.88 (11)	C15—C17—H17A	109.5
C3—C4—C5	121.33 (11)	C15—C17—H17B	109.5
C6—C5—C4	118.03 (11)	H17A—C17—H17B	109.5
C6—C5—H5	121.0	C15—C17—H17C	109.5
C4—C5—H5	121.0	H17A—C17—H17C	109.5
C5—C6—O3	118.14 (11)	H17B—C17—H17C	109.5
C5—C6—C1	123.31 (11)	C15—C18—H18A	109.5
O3—C6—C1	118.54 (10)	C15—C18—H18B	109.5
C10—C7—C9	110.38 (12)	H18A—C18—H18B	109.5
C10—C7—C8	109.03 (13)	C15—C18—H18C	109.5
C9—C7—C8	108.88 (13)	H18A—C18—H18C	109.5
C10—C7—P1	113.20 (10)	H18B—C18—H18C	109.5
C9—C7—P1	110.57 (10)	C21—C19—C22	110.54 (12)
C8—C7—P1	104.56 (10)	C21—C19—C20	109.04 (12)
C7—C8—H8A	109.5	C22—C19—C20	109.04 (13)
C7—C8—H8B	109.5	C21—C19—P2	113.53 (10)
H8A—C8—H8B	109.5	C22—C19—P2	109.00 (10)
C7—C8—H8C	109.5	C20—C19—P2	105.49 (9)
H8A—C8—H8C	109.5	C19—C20—H20A	109.5
H8B—C8—H8C	109.5	C19—C20—H20B	109.5
C7—C9—H9A	109.5	H20A—C20—H20B	109.5
C7—C9—H9B	109.5	C19—C20—H20C	109.5
H9A—C9—H9B	109.5	H20A—C20—H20C	109.5
C7—C9—H9C	109.5	H20B—C20—H20C	109.5
H9A—C9—H9C	109.5	C19—C21—H21A	109.5
H9B—C9—H9C	109.5	C19—C21—H21B	109.5
C7—C10—H10A	109.5	H21A—C21—H21B	109.5
C7—C10—H10B	109.5	C19—C21—H21C	109.5
H10A—C10—H10B	109.5	H21A—C21—H21C	109.5
C7—C10—H10C	109.5	H21B—C21—H21C	109.5
H10A—C10—H10C	109.5	C19—C22—H22A	109.5
H10B—C10—H10C	109.5	C19—C22—H22B	109.5
C12—C11—C13	111.26 (12)	H22A—C22—H22B	109.5
C12—C11—C14	108.92 (12)	C19—C22—H22C	109.5
C13—C11—C14	108.27 (12)	H22A—C22—H22C	109.5
C12—C11—P1	109.81 (10)	H22B—C22—H22C	109.5
C13—C11—P1	113.17 (10)	C2—O1—P1	114.38 (7)
C14—C11—P1	105.13 (9)	C4—O2—H2	108.4 (15)
C11—C12—H12A	109.5	C6—O3—P2	114.27 (8)
C11—C12—H12B	109.5	O1—P1—C11	100.72 (5)
H12A—C12—H12B	109.5	O1—P1—C7	100.99 (5)
C11—C12—H12C	109.5	C11—P1—C7	114.87 (6)
H12A—C12—H12C	109.5	O1—P1—Pd1	104.76 (3)
H12B—C12—H12C	109.5	C11—P1—Pd1	114.73 (5)
C11—C13—H13A	109.5	C7—P1—Pd1	117.42 (4)
C11—C13—H13B	109.5	O3—P2—C19	101.43 (6)
H13A—C13—H13B	109.5	O3—P2—C15	101.10 (6)
C11—C13—H13C	109.5	C19—P2—C15	114.50 (6)
H13A—C13—H13C	109.5	O3—P2—Pd1	104.87 (3)
H13B—C13—H13C	109.5	C19—P2—Pd1	115.77 (4)
C11—C14—H14A	109.5	C15—P2—Pd1	116.12 (5)
C11—C14—H14B	109.5	C1—Pd1—P1	80.11 (3)
H14A—C14—H14B	109.5	C1—Pd1—P2	79.73 (3)
C11—C14—H14C	109.5	P1—Pd1—P2	159.768 (12)
H14A—C14—H14C	109.5	C1—Pd1—Cl1	179.06 (4)
H14B—C14—H14C	109.5	P1—Pd1—Cl1	99.219 (13)
C17—C15—C16	110.51 (13)	P2—Pd1—Cl1	100.957 (13)
C17—C15—C18	109.05 (14)		
			
C6—C1—C2—O1	178.90 (10)	C14—C11—P1—Pd1	−31.95 (10)
Pd1—C1—C2—O1	−0.73 (16)	C10—C7—P1—O1	61.39 (11)
C6—C1—C2—C3	−0.88 (18)	C9—C7—P1—O1	−174.18 (11)
Pd1—C1—C2—C3	179.49 (9)	C8—C7—P1—O1	−57.15 (10)
O1—C2—C3—C4	−177.93 (11)	C10—C7—P1—C11	−46.00 (13)
C1—C2—C3—C4	1.85 (19)	C9—C7—P1—C11	78.44 (12)
C2—C3—C4—O2	179.12 (11)	C8—C7—P1—C11	−164.53 (10)
C2—C3—C4—C5	−0.89 (19)	C10—C7—P1—Pd1	174.54 (9)
O2—C4—C5—C6	179.06 (11)	C9—C7—P1—Pd1	−61.03 (12)
C3—C4—C5—C6	−0.93 (19)	C8—C7—P1—Pd1	56.00 (11)
C4—C5—C6—O3	−177.39 (11)	C6—O3—P2—C19	114.99 (9)
C4—C5—C6—C1	1.99 (19)	C6—O3—P2—C15	−126.92 (9)
C2—C1—C6—C5	−1.10 (18)	C6—O3—P2—Pd1	−5.84 (9)
Pd1—C1—C6—C5	178.53 (10)	C21—C19—P2—O3	65.88 (11)
C2—C1—C6—O3	178.27 (11)	C22—C19—P2—O3	−170.43 (10)
Pd1—C1—C6—O3	−2.10 (16)	C20—C19—P2—O3	−53.46 (11)
C3—C2—O1—P1	−178.98 (9)	C21—C19—P2—C15	−42.05 (12)
C1—C2—O1—P1	1.23 (14)	C22—C19—P2—C15	81.64 (12)
C5—C6—O3—P2	−175.03 (9)	C20—C19—P2—C15	−161.39 (10)
C1—C6—O3—P2	5.56 (14)	C21—C19—P2—Pd1	178.72 (8)
C2—O1—P1—C11	−120.43 (9)	C22—C19—P2—Pd1	−57.59 (11)
C2—O1—P1—C7	121.36 (9)	C20—C19—P2—Pd1	59.38 (11)
C2—O1—P1—Pd1	−1.08 (9)	C17—C15—P2—O3	−162.98 (11)
C12—C11—P1—O1	−163.04 (10)	C16—C15—P2—O3	−37.91 (12)
C13—C11—P1—O1	−38.05 (11)	C18—C15—P2—O3	79.99 (11)
C14—C11—P1—O1	79.93 (10)	C17—C15—P2—C19	−54.84 (13)
C12—C11—P1—C7	−55.49 (12)	C16—C15—P2—C19	70.22 (12)
C13—C11—P1—C7	69.50 (11)	C18—C15—P2—C19	−171.88 (11)
C14—C11—P1—C7	−172.52 (9)	C17—C15—P2—Pd1	84.24 (11)
C12—C11—P1—Pd1	85.08 (10)	C16—C15—P2—Pd1	−150.70 (10)
C13—C11—P1—Pd1	−149.93 (9)	C18—C15—P2—Pd1	−32.80 (12)

### {2,6-Bis[(di-tert-butylphosphanyl)oxy]-4-hydroxyphenyl}chloridopalladium(II) (1) Hydrogen-bond geometry (Å, º)

**Table:**

*D*—H···*A*	*D*—H	H···*A*	*D*···*A*	*D*—H···*A*
O2—H2···Cl1^i^	0.79 (2)	2.37 (2)	3.1545 (11)	174.2 (19)

Symmetry code: (i) *x*+1/2, −*y*+1/2, *z*−1/2.

### {2,6-Bis[(di-tert-butylphosphanyl)oxy]-4-hydroxyphenyl}chloridoplatinum(II) (2) Crystal data

**Table:**

[Pt(C_22_H_39_O_3_P_2_)Cl]	*F*(000) = 1280
*M**_r_* = 644.01	*D*_x_ = 1.611 Mg m^−^^3^
Monoclinic, *P*2_1_/*n*	Mo *K*α radiation, λ = 0.71073 Å
*a* = 9.7722 (8) Å	Cell parameters from 9874 reflections
*b* = 20.1562 (16) Å	θ = 2.3–30.5°
*c* = 13.9699 (11) Å	µ = 5.52 mm^−^^1^
β = 105.1634 (13)°	*T* = 150 K
*V* = 2655.9 (4) Å^3^	Prism, colourless
*Z* = 4	0.34 × 0.21 × 0.15 mm

### {2,6-Bis[(di-tert-butylphosphanyl)oxy]-4-hydroxyphenyl}chloridoplatinum(II) (2) Data collection

**Table:**

Bruker APEXII CCD diffractometer	6413 independent reflections
Radiation source: fine-focus sealed tube	5959 reflections with *I* > 2σ(*I*)
Detector resolution: 8.3333 pixels mm^-1^	*R*_int_ = 0.023
φ and ω scans	θ_max_ = 28.0°, θ_min_ = 1.8°
Absorption correction: multi-scan (SADABS; Bruker, 2014)	*h* = −8→12
*T*_min_ = 0.34, *T*_max_ = 0.50	*k* = −25→26
25029 measured reflections	*l* = −18→18

### {2,6-Bis[(di-tert-butylphosphanyl)oxy]-4-hydroxyphenyl}chloridoplatinum(II) (2) Refinement

**Table:**

Refinement on *F*^2^	0 restraints
Least-squares matrix: full	Hydrogen site location: mixed
*R*[*F*^2^ > 2σ(*F*^2^)] = 0.016	H atoms treated by a mixture of independent and constrained refinement
*wR*(*F*^2^) = 0.037	*w* = 1/[σ^2^(*F*_o_^2^) + (0.0128*P*)^2^ + 2.002*P*] where *P* = (*F*_o_^2^ + 2*F*_c_^2^)/3
*S* = 1.02	(Δ/σ)_max_ = 0.001
6413 reflections	Δρ_max_ = 0.87 e Å^−^^3^
278 parameters	Δρ_min_ = −0.84 e Å^−^^3^

### {2,6-Bis[(di-tert-butylphosphanyl)oxy]-4-hydroxyphenyl}chloridoplatinum(II) (2) Special details

**Table:**

Geometry. All esds (except the esd in the dihedral angle between two l.s. planes) are estimated using the full covariance matrix. The cell esds are taken into account individually in the estimation of esds in distances, angles and torsion angles; correlations between esds in cell parameters are only used when they are defined by crystal symmetry. An approximate (isotropic) treatment of cell esds is used for estimating esds involving l.s. planes.

### {2,6-Bis[(di-tert-butylphosphanyl)oxy]-4-hydroxyphenyl}chloridoplatinum(II) (2) Fractional atomic coordinates and isotropic or equivalent isotropic displacement parameters (Å^2^)

**Table:**

	*x*	*y*	*z*	*U*_iso_*/*U*_eq_	
C1	0.2222 (2)	0.29907 (9)	−0.06632 (13)	0.0156 (4)	
C2	0.3236 (2)	0.27501 (9)	−0.11116 (14)	0.0156 (4)	
C3	0.3826 (2)	0.31281 (9)	−0.17280 (14)	0.0162 (4)	
H3	0.4495	0.2943	−0.2038	0.019*	
C4	0.3405 (2)	0.37898 (9)	−0.18783 (14)	0.0165 (4)	
C5	0.2411 (2)	0.40605 (9)	−0.14397 (14)	0.0186 (4)	
H5	0.2139	0.4513	−0.1539	0.022*	
C6	0.1826 (2)	0.36515 (9)	−0.08529 (14)	0.0160 (4)	
C7	0.2082 (2)	0.09861 (10)	−0.10463 (16)	0.0224 (4)	
C8	0.1139 (3)	0.13295 (13)	−0.19738 (17)	0.0368 (6)	
H8A	0.1731	0.1604	−0.2285	0.055*	
H8B	0.0438	0.1610	−0.1780	0.055*	
H8C	0.0650	0.0993	−0.2446	0.055*	
C9	0.1142 (3)	0.05599 (12)	−0.0570 (2)	0.0389 (6)	
H9A	0.0616	0.0240	−0.1057	0.058*	
H9B	0.0473	0.0845	−0.0348	0.058*	
H9C	0.1736	0.0322	0.0000	0.058*	
C10	0.3175 (3)	0.05516 (11)	−0.13603 (18)	0.0315 (5)	
H10A	0.3703	0.0290	−0.0791	0.047*	
H10B	0.3835	0.0834	−0.1597	0.047*	
H10C	0.2689	0.0252	−0.1894	0.047*	
C11	0.4449 (2)	0.14387 (10)	0.08141 (15)	0.0219 (4)	
C12	0.4032 (3)	0.08751 (11)	0.14229 (18)	0.0338 (5)	
H12A	0.4785	0.0811	0.2035	0.051*	
H12B	0.3897	0.0465	0.1033	0.051*	
H12C	0.3147	0.0991	0.1589	0.051*	
C13	0.5752 (2)	0.12509 (11)	0.04526 (18)	0.0313 (5)	
H13A	0.6550	0.1151	0.1025	0.047*	
H13B	0.6005	0.1622	0.0079	0.047*	
H13C	0.5534	0.0860	0.0023	0.047*	
C14	0.4811 (3)	0.20604 (11)	0.14718 (16)	0.0282 (5)	
H14A	0.4008	0.2172	0.1740	0.042*	
H14B	0.5004	0.2432	0.1075	0.042*	
H14C	0.5650	0.1973	0.2019	0.042*	
C15	−0.1691 (2)	0.34070 (11)	−0.02288 (16)	0.0233 (4)	
C16	−0.2283 (3)	0.40901 (12)	−0.06013 (19)	0.0345 (5)	
H16A	−0.3300	0.4053	−0.0919	0.052*	
H16B	−0.1795	0.4257	−0.1083	0.052*	
H16C	−0.2133	0.4398	−0.0040	0.052*	
C17	−0.2449 (3)	0.31316 (13)	0.0515 (2)	0.0392 (6)	
H17A	−0.3454	0.3062	0.0183	0.059*	
H17B	−0.2362	0.3448	0.1061	0.059*	
H17C	−0.2018	0.2708	0.0778	0.059*	
C18	−0.1939 (3)	0.29371 (14)	−0.1125 (2)	0.0413 (6)	
H18A	−0.1673	0.2484	−0.0894	0.062*	
H18B	−0.1360	0.3080	−0.1563	0.062*	
H18C	−0.2943	0.2947	−0.1489	0.062*	
C19	0.0864 (2)	0.38351 (10)	0.15143 (15)	0.0221 (4)	
C20	0.2450 (3)	0.39706 (13)	0.16472 (19)	0.0371 (6)	
H20A	0.2850	0.4177	0.2295	0.056*	
H20B	0.2570	0.4270	0.1122	0.056*	
H20C	0.2941	0.3551	0.1609	0.056*	
C21	0.0100 (3)	0.44929 (10)	0.15415 (16)	0.0277 (5)	
H21A	−0.0888	0.4407	0.1539	0.042*	
H21B	0.0125	0.4758	0.0958	0.042*	
H21C	0.0572	0.4736	0.2145	0.042*	
C22	0.0683 (3)	0.33748 (11)	0.23488 (16)	0.0349 (6)	
H22A	0.1101	0.3584	0.2992	0.052*	
H22B	0.1162	0.2952	0.2311	0.052*	
H22C	−0.0328	0.3295	0.2276	0.052*	
Cl1	0.03655 (8)	0.17228 (3)	0.12079 (5)	0.03816 (15)	
O1	0.36834 (15)	0.20966 (6)	−0.09302 (10)	0.0177 (3)	
O2	0.39515 (17)	0.42028 (7)	−0.24662 (11)	0.0241 (3)	
O3	0.07860 (15)	0.39053 (6)	−0.04407 (10)	0.0189 (3)	
P1	0.28950 (5)	0.16756 (2)	−0.02112 (4)	0.01541 (10)	
P2	0.02527 (5)	0.33940 (2)	0.03123 (4)	0.01579 (10)	
Pt1	0.13889 (2)	0.24213 (2)	0.01970 (2)	0.01543 (3)	
H2	0.441 (3)	0.3988 (14)	−0.277 (2)	0.039 (8)*	

### {2,6-Bis[(di-tert-butylphosphanyl)oxy]-4-hydroxyphenyl}chloridoplatinum(II) (2) Atomic displacement parameters (Å^2^)

**Table:**

	*U*^11^	*U*^22^	*U*^33^	*U*^12^	*U*^13^	*U*^23^
C1	0.0183 (10)	0.0158 (8)	0.0138 (9)	0.0009 (7)	0.0064 (7)	0.0017 (6)
C2	0.0180 (10)	0.0129 (8)	0.0154 (9)	0.0025 (7)	0.0035 (7)	0.0013 (7)
C3	0.0164 (9)	0.0175 (8)	0.0170 (9)	0.0027 (7)	0.0085 (7)	0.0014 (7)
C4	0.0169 (10)	0.0180 (8)	0.0158 (9)	−0.0004 (7)	0.0067 (7)	0.0040 (7)
C5	0.0225 (10)	0.0144 (8)	0.0206 (10)	0.0029 (7)	0.0085 (8)	0.0031 (7)
C6	0.0155 (9)	0.0173 (8)	0.0167 (9)	0.0032 (7)	0.0071 (7)	−0.0005 (7)
C7	0.0268 (11)	0.0183 (9)	0.0243 (11)	−0.0033 (8)	0.0106 (9)	−0.0060 (7)
C8	0.0354 (14)	0.0417 (13)	0.0280 (13)	0.0003 (11)	−0.0015 (10)	−0.0112 (10)
C9	0.0459 (16)	0.0342 (12)	0.0454 (15)	−0.0206 (11)	0.0273 (13)	−0.0161 (11)
C10	0.0357 (14)	0.0232 (10)	0.0411 (14)	−0.0033 (9)	0.0198 (11)	−0.0124 (9)
C11	0.0255 (11)	0.0191 (9)	0.0199 (10)	0.0026 (8)	0.0037 (8)	0.0056 (7)
C12	0.0404 (14)	0.0291 (11)	0.0297 (13)	0.0012 (10)	0.0049 (11)	0.0158 (9)
C13	0.0257 (12)	0.0321 (11)	0.0343 (13)	0.0089 (10)	0.0045 (10)	0.0051 (9)
C14	0.0299 (12)	0.0302 (11)	0.0215 (11)	−0.0028 (9)	0.0012 (9)	−0.0007 (8)
C15	0.0172 (10)	0.0271 (10)	0.0264 (11)	−0.0007 (8)	0.0074 (8)	−0.0006 (8)
C16	0.0221 (12)	0.0345 (12)	0.0446 (15)	0.0055 (10)	0.0046 (10)	0.0057 (10)
C17	0.0244 (13)	0.0469 (14)	0.0515 (16)	−0.0041 (11)	0.0191 (11)	0.0086 (12)
C18	0.0283 (13)	0.0498 (15)	0.0394 (15)	0.0007 (12)	−0.0022 (11)	−0.0166 (12)
C19	0.0281 (11)	0.0186 (9)	0.0201 (10)	0.0026 (8)	0.0074 (8)	−0.0023 (7)
C20	0.0290 (13)	0.0414 (13)	0.0372 (14)	−0.0004 (11)	0.0025 (10)	−0.0145 (11)
C21	0.0392 (14)	0.0184 (9)	0.0279 (11)	0.0038 (9)	0.0131 (10)	−0.0032 (8)
C22	0.0594 (17)	0.0269 (11)	0.0195 (11)	0.0117 (11)	0.0125 (11)	0.0014 (8)
Cl1	0.0641 (4)	0.0222 (2)	0.0437 (3)	−0.0029 (3)	0.0417 (3)	0.0040 (2)
O1	0.0222 (7)	0.0135 (6)	0.0211 (7)	0.0052 (5)	0.0120 (6)	0.0042 (5)
O2	0.0329 (9)	0.0173 (6)	0.0297 (8)	0.0041 (6)	0.0217 (7)	0.0064 (6)
O3	0.0218 (7)	0.0152 (6)	0.0243 (7)	0.0046 (5)	0.0143 (6)	0.0030 (5)
P1	0.0197 (3)	0.0121 (2)	0.0163 (2)	0.00103 (18)	0.00787 (19)	0.00132 (16)
P2	0.0180 (3)	0.0148 (2)	0.0172 (2)	0.00112 (18)	0.00937 (19)	−0.00010 (17)
Pt1	0.02031 (4)	0.01324 (4)	0.01583 (4)	0.00074 (3)	0.01022 (3)	0.00099 (2)

### {2,6-Bis[(di-tert-butylphosphanyl)oxy]-4-hydroxyphenyl}chloridoplatinum(II) (2) Geometric parameters (Å, º)

**Table:**

C1—C2	1.390 (3)	C14—H14A	0.9800
C1—C6	1.393 (2)	C14—H14B	0.9800
C1—Pt1	1.9841 (18)	C14—H14C	0.9800
C2—C3	1.384 (3)	C15—C17	1.530 (3)
C2—O1	1.390 (2)	C15—C16	1.531 (3)
C3—C4	1.395 (2)	C15—C18	1.538 (3)
C3—H3	0.9500	C15—P2	1.852 (2)
C4—O2	1.372 (2)	C16—H16A	0.9800
C4—C5	1.389 (3)	C16—H16B	0.9800
C5—C6	1.387 (3)	C16—H16C	0.9800
C5—H5	0.9500	C17—H17A	0.9800
C6—O3	1.390 (2)	C17—H17B	0.9800
C7—C9	1.531 (3)	C17—H17C	0.9800
C7—C10	1.532 (3)	C18—H18A	0.9800
C7—C8	1.543 (3)	C18—H18B	0.9800
C7—P1	1.854 (2)	C18—H18C	0.9800
C8—H8A	0.9800	C19—C21	1.527 (3)
C8—H8B	0.9800	C19—C22	1.536 (3)
C8—H8C	0.9800	C19—C20	1.537 (3)
C9—H9A	0.9800	C19—P2	1.855 (2)
C9—H9B	0.9800	C20—H20A	0.9800
C9—H9C	0.9800	C20—H20B	0.9800
C10—H10A	0.9800	C20—H20C	0.9800
C10—H10B	0.9800	C21—H21A	0.9800
C10—H10C	0.9800	C21—H21B	0.9800
C11—C13	1.535 (3)	C21—H21C	0.9800
C11—C12	1.537 (3)	C22—H22A	0.9800
C11—C14	1.539 (3)	C22—H22B	0.9800
C11—P1	1.857 (2)	C22—H22C	0.9800
C12—H12A	0.9800	Cl1—Pt1	2.3907 (5)
C12—H12B	0.9800	O1—P1	1.6514 (13)
C12—H12C	0.9800	O2—H2	0.82 (3)
C13—H13A	0.9800	O3—P2	1.6514 (13)
C13—H13B	0.9800	P1—Pt1	2.2781 (5)
C13—H13C	0.9800	P2—Pt1	2.2796 (5)
			
C2—C1—C6	116.32 (16)	C16—C15—C18	108.19 (19)
C2—C1—Pt1	121.64 (13)	C17—C15—P2	110.18 (16)
C6—C1—Pt1	122.03 (14)	C16—C15—P2	114.05 (15)
C3—C2—O1	118.22 (16)	C18—C15—P2	104.48 (15)
C3—C2—C1	123.37 (17)	C15—C16—H16A	109.5
O1—C2—C1	118.41 (16)	C15—C16—H16B	109.5
C2—C3—C4	117.77 (17)	H16A—C16—H16B	109.5
C2—C3—H3	121.1	C15—C16—H16C	109.5
C4—C3—H3	121.1	H16A—C16—H16C	109.5
O2—C4—C5	116.91 (16)	H16B—C16—H16C	109.5
O2—C4—C3	121.65 (17)	C15—C17—H17A	109.5
C5—C4—C3	121.45 (17)	C15—C17—H17B	109.5
C6—C5—C4	118.14 (17)	H17A—C17—H17B	109.5
C6—C5—H5	120.9	C15—C17—H17C	109.5
C4—C5—H5	120.9	H17A—C17—H17C	109.5
C5—C6—O3	119.03 (16)	H17B—C17—H17C	109.5
C5—C6—C1	122.91 (17)	C15—C18—H18A	109.5
O3—C6—C1	118.06 (16)	C15—C18—H18B	109.5
C9—C7—C10	110.27 (18)	H18A—C18—H18B	109.5
C9—C7—C8	108.8 (2)	C15—C18—H18C	109.5
C10—C7—C8	108.90 (19)	H18A—C18—H18C	109.5
C9—C7—P1	110.74 (15)	H18B—C18—H18C	109.5
C10—C7—P1	113.09 (15)	C21—C19—C22	110.59 (18)
C8—C7—P1	104.80 (14)	C21—C19—C20	109.09 (18)
C7—C8—H8A	109.5	C22—C19—C20	109.2 (2)
C7—C8—H8B	109.5	C21—C19—P2	113.26 (15)
H8A—C8—H8B	109.5	C22—C19—P2	108.89 (14)
C7—C8—H8C	109.5	C20—C19—P2	105.68 (14)
H8A—C8—H8C	109.5	C19—C20—H20A	109.5
H8B—C8—H8C	109.5	C19—C20—H20B	109.5
C7—C9—H9A	109.5	H20A—C20—H20B	109.5
C7—C9—H9B	109.5	C19—C20—H20C	109.5
H9A—C9—H9B	109.5	H20A—C20—H20C	109.5
C7—C9—H9C	109.5	H20B—C20—H20C	109.5
H9A—C9—H9C	109.5	C19—C21—H21A	109.5
H9B—C9—H9C	109.5	C19—C21—H21B	109.5
C7—C10—H10A	109.5	H21A—C21—H21B	109.5
C7—C10—H10B	109.5	C19—C21—H21C	109.5
H10A—C10—H10B	109.5	H21A—C21—H21C	109.5
C7—C10—H10C	109.5	H21B—C21—H21C	109.5
H10A—C10—H10C	109.5	C19—C22—H22A	109.5
H10B—C10—H10C	109.5	C19—C22—H22B	109.5
C13—C11—C12	111.28 (18)	H22A—C22—H22B	109.5
C13—C11—C14	108.47 (18)	C19—C22—H22C	109.5
C12—C11—C14	108.90 (18)	H22A—C22—H22C	109.5
C13—C11—P1	113.03 (15)	H22B—C22—H22C	109.5
C12—C11—P1	109.56 (15)	C2—O1—P1	115.03 (11)
C14—C11—P1	105.35 (14)	C4—O2—H2	110.2 (19)
C11—C12—H12A	109.5	C6—O3—P2	114.89 (11)
C11—C12—H12B	109.5	O1—P1—C7	101.18 (8)
H12A—C12—H12B	109.5	O1—P1—C11	100.58 (8)
C11—C12—H12C	109.5	C7—P1—C11	114.96 (9)
H12A—C12—H12C	109.5	O1—P1—Pt1	104.36 (5)
H12B—C12—H12C	109.5	C7—P1—Pt1	116.95 (7)
C11—C13—H13A	109.5	C11—P1—Pt1	115.35 (7)
C11—C13—H13B	109.5	O3—P2—C15	101.07 (9)
H13A—C13—H13B	109.5	O3—P2—C19	101.36 (8)
C11—C13—H13C	109.5	C15—P2—C19	114.56 (10)
H13A—C13—H13C	109.5	O3—P2—Pt1	104.54 (5)
H13B—C13—H13C	109.5	C15—P2—Pt1	116.74 (7)
C11—C14—H14A	109.5	C19—P2—Pt1	115.39 (7)
C11—C14—H14B	109.5	C1—Pt1—P1	80.54 (5)
H14A—C14—H14B	109.5	C1—Pt1—P2	80.20 (5)
C11—C14—H14C	109.5	P1—Pt1—P2	160.676 (17)
H14A—C14—H14C	109.5	C1—Pt1—Cl1	178.95 (6)
H14B—C14—H14C	109.5	P1—Pt1—Cl1	99.023 (19)
C17—C15—C16	110.49 (19)	P2—Pt1—Cl1	100.257 (19)
C17—C15—C18	109.2 (2)		
			
C6—C1—C2—C3	−0.9 (3)	C8—C7—P1—Pt1	55.81 (16)
Pt1—C1—C2—C3	178.98 (15)	C13—C11—P1—O1	−38.88 (16)
C6—C1—C2—O1	178.89 (16)	C12—C11—P1—O1	−163.60 (15)
Pt1—C1—C2—O1	−1.2 (2)	C14—C11—P1—O1	79.40 (15)
O1—C2—C3—C4	−177.85 (17)	C13—C11—P1—C7	68.85 (17)
C1—C2—C3—C4	2.0 (3)	C12—C11—P1—C7	−55.87 (18)
C2—C3—C4—O2	179.05 (18)	C14—C11—P1—C7	−172.87 (14)
C2—C3—C4—C5	−1.0 (3)	C13—C11—P1—Pt1	−150.45 (13)
O2—C4—C5—C6	179.09 (18)	C12—C11—P1—Pt1	84.83 (15)
C3—C4—C5—C6	−0.8 (3)	C14—C11—P1—Pt1	−32.18 (16)
C4—C5—C6—O3	−177.37 (17)	C6—O3—P2—C15	−127.19 (14)
C4—C5—C6—C1	2.0 (3)	C6—O3—P2—C19	114.69 (14)
C2—C1—C6—C5	−1.1 (3)	C6—O3—P2—Pt1	−5.57 (14)
Pt1—C1—C6—C5	178.99 (15)	C17—C15—P2—O3	−163.37 (16)
C2—C1—C6—O3	178.22 (17)	C16—C15—P2—O3	−38.44 (17)
Pt1—C1—C6—O3	−1.7 (2)	C18—C15—P2—O3	79.49 (17)
C3—C2—O1—P1	−178.45 (14)	C17—C15—P2—C19	−55.30 (19)
C1—C2—O1—P1	1.7 (2)	C16—C15—P2—C19	69.62 (18)
C5—C6—O3—P2	−175.52 (15)	C18—C15—P2—C19	−172.45 (16)
C1—C6—O3—P2	5.1 (2)	C17—C15—P2—Pt1	83.99 (16)
C2—O1—P1—C7	120.46 (14)	C16—C15—P2—Pt1	−151.09 (14)
C2—O1—P1—C11	−121.21 (13)	C18—C15—P2—Pt1	−33.16 (18)
C2—O1—P1—Pt1	−1.38 (13)	C21—C19—P2—O3	66.03 (17)
C9—C7—P1—O1	−173.96 (16)	C22—C19—P2—O3	−170.48 (15)
C10—C7—P1—O1	61.70 (17)	C20—C19—P2—O3	−53.34 (16)
C8—C7—P1—O1	−56.78 (16)	C21—C19—P2—C15	−41.87 (19)
C9—C7—P1—C11	78.67 (19)	C22—C19—P2—C15	81.62 (17)
C10—C7—P1—C11	−45.66 (19)	C20—C19—P2—C15	−161.23 (15)
C8—C7—P1—C11	−164.15 (15)	C21—C19—P2—Pt1	178.29 (13)
C9—C7—P1—Pt1	−61.38 (18)	C22—C19—P2—Pt1	−58.23 (17)
C10—C7—P1—Pt1	174.29 (13)	C20—C19—P2—Pt1	58.92 (16)

### {2,6-Bis[(di-tert-butylphosphanyl)oxy]-4-hydroxyphenyl}chloridoplatinum(II) (2) Hydrogen-bond geometry (Å, º)

**Table:**

*D*—H···*A*	*D*—H	H···*A*	*D*···*A*	*D*—H···*A*
O2—H2···Cl1^i^	0.82 (3)	2.38 (3)	3.1874 (16)	170 (3)

Symmetry code: (i) *x*+1/2, −*y*+1/2, *z*−1/2.
